# Can nonresponse bias and known methodological differences explain the large discrepancies in the reported prevalence rate of violence found in Swedish studies?

**DOI:** 10.1371/journal.pone.0216451

**Published:** 2019-05-09

**Authors:** Johanna Simmons, Katarina Swahnberg

**Affiliations:** 1 Department of Acute Internal Medicine and Geriatrics, and Department of Clinical and Experimental Medicine, Linköping University, Linköping, Sweden; 2 Department of Health and Caring Sciences, Faculty of Health and Life Sciences, Linnaeus University, Kalmar, Sweden; Middlesex University, UNITED KINGDOM

## Abstract

**Introduction:**

The reported prevalence rate of violence varies considerably between studies, even when conducted in similar populations. The reasons for this are largely unknown. This article considers the effects of nonresponse bias on the reported prevalence rate of interpersonal violence. We also single out violence perpetrated in intimate relationships and compare our results to previous Swedish studies. The aim was to explore the reasons for the large discrepancies in the prevalence rates found between studies.

**Material and method:**

This is a cross sectional study of a random population sample. The NorVold Abuse Questionnaire (NorAQ), covering emotional, physical, and sexual violence, was answered by 754 men (response rate 35%) and 749 women (response rate 38%). Nonresponse bias was investigated in six ways, e.g., findings were replicated in two samples and we explored non-responders’ reasons for declining participation. Also, the prevalence rate of intimate partner violence was compared to four previous studies conducted in Sweden, considering the methodological differences.

**Results and discussion:**

The only evidence of nonresponse bias found was for differences between the sample and the background population concerning the sociodemographic characteristics. However, the magnitude of that effect is bleak in comparison with the large discrepancies found in the prevalence rates between studies concerning intimate partner violence, e.g., emotional violence women: 11–41% and men: 4–37%; sexual and/or physical violence women: 12–27% and men: 2–21%. Some of the reasons behind these differences were obvious and pertained to differences in the definition and operationalization of violence. However, a considerable proportion of the difference could not easily be accounted for.

**Conclusion:**

It is not reasonable that so little is known about the large discrepancies in the prevalence rate for what is supposedly the same concept, i.e., intimate partner violence. This study is a call for more empirical research on methods to investigate violence.

## Introduction

The reported prevalence of violence varies considerably between studies. For example, in the U.S., the prevalence rates of violence in otherwise similar community samples have been reported between 2% and 60% [1]. Such discrepancies lower confidence in the research field and cause problems for researchers and policy makers, especially when evaluating the effect of interventions to reduce violence. This study focuses on the methodology in violence research and seeks to explore if nonresponse bias and known methodological differences can explain the discrepancies in the prevalence rates reported in Swedish studies.

The most commonly reported indicator of nonresponse bias is the response rate. However, it does not determine the magnitude of the nonresponse bias. Hence, it is important to assess the extent to which nonresponse is associated with the studied exposure and/or outcome [2–4]. Several ways to assess the level of nonresponse bias have been suggested [4]. One way is to replicate the findings in a new sample. Another way is to compare the results of the study to known characteristics of the population, most commonly sociodemographic characteristics. Yet another approach is to compare the early and late responders using a wave analysis. This is based on the theory that those responding late have special characteristics and that time to respond can be used to estimate nonresponse bias [4]. Also, it is possible that different modes of data collection (e.g., paper or web) attract different responders and thereby affect the results. Thus, an analysis of the technical methodology might expose the nonresponse bias.

Non-responders’ reasons for declining participation are the most elusive. Participation may be higher among those with the studied exposure, due to an increased interest in the study [2, 4]. If the exposure is stigmatized, however, participation may be lower among the exposed [2]. Using questions within the survey to assess the level of interest among respondents have been suggested as a potential indicator of nonresponse bias. One variant of this would be to examine respondents’ willingness to participate in a follow-up study [4].

To investigate the reliability of a study, it is also important to benchmark ones results with previously published data [4]. This is difficult in violence research, considering the divergent prevalence rates reported in studies. Therefore, methodological differences between studies must be considered when comparing the results. One factor to consider is how the researcher conceptualizes, understands, and operationalizes the term violence. This will inevitably affect the results [5, 6]. Discrepancies are evident, for example, in the gender symmetry debate. Some studies report that men and women are victims and perpetrators of intimate partner violence to the same extent in intimate partner relationships, i.e., gender symmetry [7, 8]. This finding, however, has been disputed by many and likely pertains to the research methodology and decontextualization of violence [1, 9–14].

Another example of how important conceptualization is concerns the co-occurrence of violence. Victims of one form of violence are often victims of other forms of violence [6, 15–18]. This can be classified as polyvictimization and was first studied concerning children [19, 20]. It has received increased attention and is now also considered in studies involving adults and the older adults [15, 21]. However, much research in the field is still conducted in specialized fields, e.g., intimate partner violence, sexual violence, bullying, etc. Such compartmentalization affects the methodology and the results. For example, when only intimate partner violence is studied, it is common to use a perpetrator specific behavioral checklist. Statements about the partner’s behavior are used, e.g., “my partner slapped me.” This can be contrasted against instruments using non-partner specific questions e.g., “has someone slapped you?,” followed by a question about who the perpetrator was, which is then used to report the rates of intimate partner violence. The difference might be perceived as small, but has been found to have a significant effect on the reported prevalence rates of intimate partner violence [10].

Definitions and operationalizations also affect the risk of false positive and false negative answers. In research on intimate partner violence, underreporting is often assumed to be the larger problem. This is either because the subject is sensitive, because severely victimized persons are unlikely to participate, or due to recall bias of less severe forms of violence [14, 22–24]. Potential false positives have only been investigated in a few studies. Some victims have been found to include playful violence and mock violence when answering surveys about intimate partner violence [10, 25, 26].

### Aim

Our overall aim was to investigate the potential methodological reasons for the diverging prevalence rates of violence in different studies. This was achieved by assessing the nonresponse bias in a study of interpersonal violence, i.e., violence perpetrated by any kind of perpetrator. We also singled out violence perpetrated by intimate partners and compared our results with four previous Swedish studies concerning intimate partner violence, reporting considerably different prevalence rates [23, 24, 27, 28]. Our specific aims were:

1. To investigate the effects of nonresponse bias on the prevalence of interpersonal violence in a random population sample by:
  - Replicating the findings between two studies using the same instrument and the same background population.
  - Investigating the sociodemographic characteristics of non-respondents and how sample characteristics influenced the prevalence rate.
  - Wave analysis, i.e., investigating the differences in the prevalence rates, depending on whether the respondents answered promptly or after one or two reminders.
  - Investigating if there were differences in the prevalence rates, depending on whether the respondents chose to answer by paper or online.
  - Estimating the interest level among the non-respondents by investigating associations between willingness to participate in a follow-up study and exposure to violence.
  - Investigating the reasons provided by non-respondents for declining participation.
2. To investigate the prevalence of intimate partner violence and compare it to the results of previous Swedish studies, in relation to the methodological differences.

## Material and methods

### Aim one: Nonresponse bias in violence research

#### Procedure and sample

In 2012, a questionnaire was sent to 2,200 men and 2,000 women, aged 25–85 randomly selected from the population of the county of Östergötland in Sweden. Two reminders were used, and 79 forms were returned to the sender because of delivery problems or other practical issues. Respondents were given the option of choosing between an online and a paper format. In total, 754 men (35%) and 749 women (38%) returned a completed questionnaire either on paper (n = 1,320) or online (n = 183). In addition, 359 women (18%) and 321 men (15%) men returned only the last page of the survey, declining participation. Among those, 450 (66%) answered a question about their reasons for non-participation.

Previously collected data, using the same instrument in the same general population, was used to replicate the findings (aim 1A). That data were collected in 2000 (women, n = 1,168 response rate 61%) and in 2007 (men, n = 2,924 response rate 50%) and are hereinafter referred to as the “old sample” and the “old data collection,” while the 2012 data collection is referred to as the “new sample” and the “new data collection.” The procedure for the old data collections has been thoroughly described elsewhere and is almost identical to the 2012 procedure previously described [29, 30].

#### Instrument

The NorVold Abuse Questionnaire (NorAQ) was used to collect the data in both samples. NorAQ was originally developed for a multicenter study among women visiting gynecology clinics in the Nordic countries [31]. Later, a male version was developed, and both versions have been validated with satisfying results [32, 33]. NorAQ covers experiences of sexual, physical, and emotional violence. The questions are presented in conjunction with the second aim. In the new data collection, the response categories were slightly modified. In the original version, the possible answers were “no,” “yes as a child (<18 years),” “yes as an adult (>18 years),” and “yes both as an adult and as a child.” This was followed by one question for each form of violence (not each question) about who the perpetrator was. In the new data collection, each question was answered with “yes” or “no” and followed with a question about the perpetrator. The old data collection also included a question about mild physical violence (e.g., slapping or holding firmly) but it had low validity; therefore, it was omitted from the new data collection. Respondents in the old data collections, answering affirmatively to that question but “no” to the questions about moderate or severe physical violence were therefore considered as non-victims of physical violence.

Respondents were also asked about their willingness to participate in follow-up studies, using either a questionnaire or an interview, as well as about feelings of malaise when completing the survey.

#### Statistical analyses

The significance level in all of the analysis was set to 95%.

To explore the effects of the nonresponse bias, the following analyses were conducted: A) Replicate findings: the proportion of respondents reporting each severity of violence in the old and the new samples was compared using chi-square test. The two data collections included respondents within different age ranges, but only respondents within the overlapping age range (25–65 years) were included in the analysis for aim 1A. B) An analysis of nonresponse in the new sample was conducted by comparing the socioeconomic background with official statistics regarding the background population [34]. To explore the potential effect of nonresponse on prevalence rates, binary logistic regression analyses were performed. Socioeconomic characteristics were used as independent factors, and reporting each form of violence (yes/no) was used as a dependent factor. C) Pearson’s chi square test was used to investigate the differences in the prevalence rate between those who answered the survey promptly as opposed to those who answered after one or two reminders. D) Pearson’s chi square test was used to investigate the differences in the prevalence rates between those answering by paper and those answering online. E) Chi square test was used to investigate if respondents exposed to violence were more willing to participate in the follow-up studies than those who were not exposed. Among victims, we also investigated if feeling malaise when answering the NorAQ was associated with a willingness to participate in the follow-up studies. For the latter analysis, male and female respondents were merged to achieve a higher statistical power. F) Descriptive statistics were used to investigate the reasons for non-participation, given by those returning only the last page of the survey.

### Aim two: Discrepancies in the reported prevalence rate of intimate partner violence

For aim two, the new data collection was used, but only violence perpetrated by an intimate partner was included. The results were compared to four previously published studies concerning intimate partner violence in Sweden. Data for the four studies were gathered from their respective publications [23, 24, 27, 28]. In this paper, emotional as well as psychological violence and controlling behaviors will be referred to as emotional violence.

### Ethical considerations

The old and the new data collection was approved by the regional ethical review board in Linköping (registration number 37–07 and 194–31). Asking about violence can be a sensitive topic, triggering memories and flashbacks for victims. Contact information for an independent therapist was therefore provided in the letter accompanying the questionnaire.

## Results

### 1A –Replicate findings

There was no statistically significant difference in the overall prevalence rate of each kind of violence between the samples. However, when comparison of the prevalence rate for each degree of severity of violence was calculated, mild sexual violence was more common in the new female sample (4.5% vs 2.2%, p = 0.02), and moderate emotional violence was more common in the new male sample 4.0% vs 2.3%, p = 0.03) (Table 1).

**Table 1:** Comparisons of the prevalence (%) rate of violence victimization in the old and the new sample.

	Women			Men		
	2001 (n = 1021)	2012 (n = 542)		2007 (n = 2596)	2012 (n = 504)	
**Physical**						
No, mild	79.2	79.6		65.4	66.3	
Moderate	14.1	14.9		25.2	21.6	
Severe	6.7	5.5		9.5	12.1	
**Emotional**						
No	79.9	78.7		84.1	83.8	
Mild	6.5	8.5		6.7	6.6	
Moderate	3.9	5.0		**2.3**	**4.0**	*
Severe	9.7	7.8		6.9	5.6	
**Sexual**						
No	84.1	80.4		95.8	95.6	
Mild	**2.3**	**4.5**	*	1.2	1.6	
Moderate	6.1	7.4		1.8	2.2	
Severe	7.5	7.7		1.2	0.6	

Note

* = p<0.05.

Comparison only includes respondents in the overlapping age range of 25–65 years old

### 1B - Non-responders in the new sample

Details regarding the differences in the sociodemographic characteristics between the new sample and the background population can be found in Table 2. The youngest (25–34) and the oldest (75–84) women are under sampled, as are younger men. Swedish born respondents as well as women with high income and higher education are over sampled. (Table 2)

**Table 2:** Comparisons of the sociodemographic characteristics between the new sample and the background population.

		Women			Men		
		2012	SCB		2012	SCB	
		%	%		%	%	
Age group	25–34	**13.1**	**17.4**	*	**12.7**	**19.1**	**
	35–44	19.6	18.6		**13.1**	**19.3**	**
	45–54	19.9	19.1		18.3	19.7	
	55–64	21.0	18.3		**21.1**	**18.1**	*
	65–74	**19.7**	**16.5**	*	**23.2**	**15.9**	**
	75–84	**6.7**	**10.1**	*	**11.7**	**7.9**	**
Marital status	Married	**56.5**	**50.8**	*	**62.4**	**50.0**	**
	Unmarried	**43.5**	**49.2**		**37.6**	**50.0**	
Education	≤ 9 years	**15.4**	**20.5**	**	24.0	21.2	
	10–12 years	**38.5**	**43.6**	*	**42.5**	**47.4**	*
	>13 years	**46.2**	**35.9**	**	33.5	31.4	
Occupation	Working	62.3	64.6		68.1	71.2	
	Not working	37.7	35.4		31.9	28.8	
Income	0–19 900	**45.1**	**53.9**	**	35.5	34.6	
	20–29 900	**37.9**	**33.2**	*	32.8	31.4	
	30–49 900	**15.2**	**11.5**	**	25.4	27.6	
	>50 000	1.8	1.4		6.3	6.4	
Country of birth	Sweden	**91.1**	**85.3**	**	**90.4**	**86.0**	**
	Other	**8.9**	**14.7**		**9.6**	**14.0**	

Note:

* = p<0.05

** p = <0.001

Concerning occupation, SCB data were only available up to 74 years of age; therefore, the comparison only includes respondents between 25–74. Concerning the country of birth, the SCB does not have an upper age limit.

For women, young age was associated with reporting sexual violence (25–34 years: Adj OR 14.6 CI 1.8–116.9). Being unmarried was associated with reporting all three kinds of violence (emotional: Adj OR 2.7 95% CI 1.8–4.1, Physical: Adj OR 2.3 95% CI 1.5–3.5, and Sexual: Adj OR 1.6 95% CI 1.0–2.3). For men, being born in another country was associated with reporting emotional (Adj OR 2.1 95% CI 1.1–4.1) and physical violence (Adj OR 2.1 95% CI 1.2–3.6). Young men were more likely to report experiences of physical violence (e.g., 25–34 years Adj OR 3.5 95% CI 1.4–8.8) (Table 3).

**Table 3:** Odds of reporting violence, depending on the sociodemographic characteristics.

		Women (n = 749)									Men (n = 754)								
		Emotional			Physical			Sexual			Emotional			Physical			Sexual		
		OR	95%CI		OR	95%CI		OR	95%CI		OR	95%CI		OR	95%CI		OR	95%CI	
**Age group**	25–34	3.5	0.9	13.6	1.9	0.5	6.8	**14.6**	**1.8**	**116.9**	1.7	0.6	5.3	**3.5**	**1.4**	**8.8**	0.9	0.1	6.5
	35–44	**6.3**	**1.7**	**23.6**	**4.0**	**1.2**	**13.2**	**10.4**	**1.3**	**83.4**	2.7	0.8	8.4	**4.4**	**1.7**	**11.3**	2.7	0.4	17.3
	45–54	**5.1**	**1.3**	**19.5**	3.2	0.9	10.9	**14.6**	**1.8**	**118.3**	2.5	0.9	7.4	**3.8**	**1.5**	**9.3**	2.5	0.4	14.8
	55–64	2.8	0.7	10.8	**3.3**	**1.0**	**10.9**	**9.7**	**1.2**	**77.0**	**3.2**	**1.2**	**8.5**	1.7	0.7	4.1	0.9	0.2	5.3
	65–74	2.4	0.7	8.6	2.4	0.8	7.6	**7.9**	**1.0**	**60.9**	1.4	0.6	3.7	1.8	0.8	3.9	0.2	0.0	1.6
	75–84	1.0			1.0			1.0			1.0			1.0			1.0		
**Civil state**	Married	1.0			1.0			1.0			1.0			1.0			1.0		
	Not married	**2.7**	**1.8**	**4.1**	**2.3**	**1.5**	**3.5**	**1.6**	**1.0**	**2.3**	**1.9**	**1.2**	**2.9**	1.2	0.9	1.8	0.5	0.2	1.3
**Education**	< 9 years	1.0			1.0			1.0			1.0			1.0			1.0		
	10–12 years	1.3	0.6	2.6	0.8	0.4	1.5	1.1	0.5	2.3	1.3	0.7	2.5	1.2	0.7	2.0	2.4	0.5	12.4
	>13 years	1.9	0.9	4.0	1.1	0.6	2.0	1.5	0.7	3.1	1.7	0.9	3.4	1.2	0.7	2.1	**6.1**	**1.2**	**31.3**
**Occupation**	Working	1.5	0.8	2.8	1.1	0.6	2.0	0.8	0.4	1.4	0.7	0.3	1.3	0.8	0.4	1.4	0.5	0.1	2.1
	Not working	1.0			1.0			1.0			1.0			1.0			1.0		
**Income**	0–19900	3.4	0.6	18.0	2.0	0.4	10.1	1.4	0.3	6.9	3.4	0.9	13.1	0.9	0.4	2.1	4.8	0.4	51.8
(SEK)	20–29900	1.3	0.3	6.4	1.3	0.3	6.5	1.2	0.2	5.7	2.9	0.8	10.5	1.2	0.6	2.5	5.2	0.6	45.7
	30–49900	0.9	0.2	4.8	0.6	0.1	3.2	1.1	0.2	5.7	1.8	0.5	6.7	1.3	0.6	2.7	1.9	0.2	17.2
	>50000	1.0			1.0			1.0			1.0			1.0			1.0		
**Country of birth**	Sweden	1.0			1.0			1.0			1.0			1.0			1.0		
	Other	1.0	0.5	2.0	1.4	0.7	2.8	0.9	0.4	1.9	**2.2**	**1.1**	**4.0**	**2.1**	**1.2**	**3.6**	1.4	0.4	4.4

### 1C- Wave analysis

Almost half (47.9%) of the respondents answered the original dispatch, 33% responded after one, and 18.3% after two reminders. Women were more inclined to respond without any reminders and men after two reminders. Newly retired men and women (age 65–74 years) were more likely to respond promptly, while younger and middle age people were more likely to need reminders. The prevalence rate did not differ between those answering promptly and those needing reminders (emotional p = 0.25, physical p = 0.21, and sexual p = 0.82).

### 1D- Prevalence rate depending on the mode of response (paper/web)

In total, 87.9% responded via paper and 12.1% via the web. Respondents choosing to answer via the web reported a higher prevalence of physical violence (32% vs. 24%, p = 0.01). However, men and younger people were overrepresented among those answering online. Therefore, a binary logistic regression was calculated, including sex, age, and mode of response as independent variables. In the adjusted model, the association between physical violence and mode of response did not remain (p = 0.36).

### 1E –Willingness to participate in further studies

No statistically significant difference was found between victims and non-victims concerning their willingness to participate in a follow-up study. Among men and women exposed to violence, the following trend was found; those who reported none or moderate feelings of unease were more likely to be willing to participate in a follow-up study using a questionnaire, but victims reporting intense feelings of malaise were less willing (p = 0.06) (Table 4).

**Table 4:** Willingness to participate in a follow-up questionnaire study and/or interview study.

		New questionnaire					Interview				
		Yes		No			Yes		No		
		N	%	N	%	p-value	N	%	N	%	p-value
**Women**											
Exposed	Yes	176	35.2	67	28.8	0.08	138	36.1	103	30.1	0.09
	No	324	64.8	166	71.2		244	63.9	239	69.9	
**Men**											
Exposed	Yes	179	36.3	86	34.1	0.51	145	36.2	119	35.1	0.77
	No	310	63.4	166	65.9		256	63.8	220	64.9	
**Men and women exposed to violence**											
Feeling	None	257	73.2	118	77.6	0.06	210	75.0	163	74.1	0.41
malaise	Moderate	89	25.4	28	18.4		66	23.6	50	22.7	
	Intense	5	1.4	6	3.9		4	1.4	7	3.2	

### 1F - Reasons for declining participation

**Fig 1:**
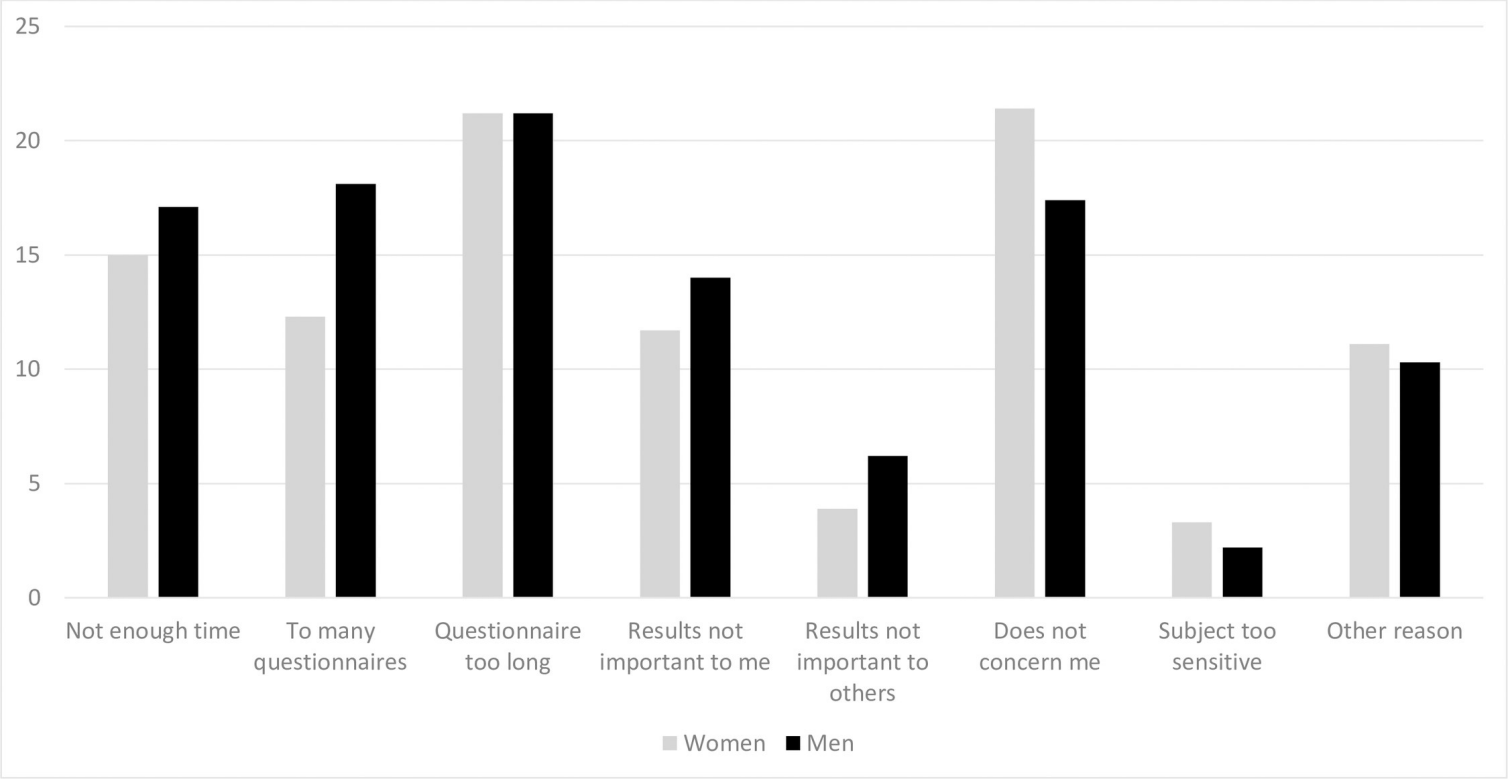
Reasons for declining participation (percentages).

Reasons for declining participation are presented in Fig 1. Most common reasons were answers indicating a lack of interest (“does not concern me”) or being tired of surveys in general (“questionnaire too long,” “I receive too many questionnaires,” “I don’t have enough time”). Very few (2.8%, n = 19) indicated that the subject was too sensitive. A considerable proportion (10.7%, n = 73) stated that they had “other reasons” for not participating. Most commonly, they reported not being well enough, too old, or something similar. The only statistically significant difference for declining participation between the sexes was that more men (18.1% n = 58) than women (12.3% n = 44) reported that they had received too many questionnaires (p = 0.03) (Fig 1).

### Aim two–Discrepancies in the reported prevalence of intimate partner violence

The items used to measure different forms of intimate partner violence in the respective studies are presented in Table 5. Survey characteristics of the different studies are presented in Table 6. The lifetime prevalence of any form of intimate partner violence was found to be 16% for women and 5% for men in this study. Prevalence rates for this and the other studies are presented in Table 7.

**Table 5:** Items that constitute exposure to violence in different Swedish studies.

**Emotional violence**	
**NorAQ**	Have you experienced 1) anybody systematically and for a long period trying to repress, degrade, or humiliate you? 2) by threat or force trying to limit your contacts with others or totally control what you may and may not do? 3) living in fear because somebody systematically and for a long period has threatened you or somebody close to you?
**NCK study:**	Has someone done any of the following to you? Your partner (or former partner) systematically and repeatedly 1) belittled, insulted, degraded, or in other ways offended or oppressed you with words? 2) dominated you or decided for you: whom you may see, how much money you may have, when you can go out, what clothes you should wear, etcetera? 3) threatened to hurt himself/herself or your children or to take the children and leave you, or to break your valued possessions or share your secrets, etcetera? 4) Someone systematically and repeatedly bullied, offended, or harassed you (e.g., relatives, at your work, in your school, or something similar)?
**Extended** **NTU:**	Has your partner: 1) systematically offended, insulted, belittled, or humiliated you? 2) not allowed you to have contact with a specific person, stopped you from leaving your home, or in similar ways tried to decide what you can do? 3) threatened you (e.g., threatened to hurt you or other persons or destroy objects) so that you felt scared? 4) followed or harassed you on repeated occasions (e.g., unwanted e-mail, text messages, phone calls, visits, or similar)?
**Lövestad study:** **(CBS)**	My partner: 1) tried to restrict the time I spent with my family/friends, 2) wanted to know where I went and who I spoke to when not together, 3) tried to limit my activities outside the relationship, 4) felt suspicious and jealous of me, and 5) tried to control my activities.
**Nybergh study:** **(VAWI)**	My partner: 1) insulted me in a way that made me feel bad, 2) belittled and humiliated me in front of others, 3) tried to scare and terrorize me on purpose, and 4) threatened to hurt me or someone I care about.
**Physical violence**	
**NorAQ**	Have you experienced anybody 1) hitting you with his/her fist(s) or with a hard object, kicking you, pushing you violently, giving you a beating, thrashing you, or doing anything similar to you? 2) threatening your life by, for instance, trying to strangle you, showing a weapon or knife, or by any other similar act?
**NCK study:**	After your 18^th^ birthday, has someone done any of the following to you? 1) threatened to hurt you with physical violence? 2) hit you with an open palm, pulled your hair, pushed you, or shook you so that it hurt? 3) hit you with his/her fist or a hard object, kicked you, or grabbed you in a stranglehold? 4) hurt you with a knife or a firearm? And 5) used other kinds of physical violence towards you?
**Extended** **NTU:**	Has your partner: 1) grabbed you in a violent way, pushed you, thrown something hard against you, slapped you, or something similar? 2) hurt you with an object, hit you with his/her fist, kicked you, or something similar?
**Lövestad study:** **(CTS2)**	My Partner: 1) threw something at me, 2) twisted my arm or hair, 3) pushed or shoved me, 4) used a knife or tool, 5) hit me with something that could hurt, 6) choked me, 7) slammed me against a wall, 8) beat me up, 9) grabbed me, 10) slapped me, 11) burned or scalded me, and 12) kicked me.
**Nybergh study:** **(VAWI)**	My partner: 1) pushed or shoved me, 2) threw something that could have hurt me, 3) hit me with his/her fist or with some other object, 4) kicked and dragged me and beat me up, 5) choked me or burnt me on purpose, and 6) hurt me with a knife, a gun, or other weapon.
**Sexual violence**	
**NorAQ**	1) Have you been sexually humiliated, e.g., by being forced to watch a pornographic movie or similar against your will, forced to participate in a pornographic movie or similar, forced to show your body naked, or forced to watch when somebody else showed his/her body naked? Has anybody against your will 2) touched parts of your body other than the genitals in a ‘sexual way’ or forced you to touch other parts of his or her body in a ‘sexual way’? 3) touched your genitals, used your body to satisfy him/herself sexually or forced you to touch anybody else’s genitals 4) put his penis into your vagina, mouth or rectum or tried any of this; put in or tried to put an object or other part of the body into your vagina, mouth or rectum?
**NCK study:**	After your 18^th^ birthday, has someone done any of the following to you? 1) forced you or 2) tried to force you to have intercourse (oral, vaginal, anal) or perform any other similar sexual act (e.g., masturbation) by threatening to or actually using physical force? 3) forced or tried to force you to have some kind of sexual activity when you were defenseless because you were sleeping, sick or affected by drugs or alcohol? 4) against your will touched or tried to touch your body in a sexual way (for example, caressed, held, hugged, kissed, groped) or made you touch him/her in a sexual way?
**Extended** **NTU:**	Has your partner: 1) subjected you to, or involved you in a sexual act against your will? 2) forced you to engage in some kind of sexual act by threatening you, grabbing you or taking advantage of you when you were drunk, or similar?
**Lövestad study:** **(CTS2)**	My partner: 1) made me have sex without a condom, 2) used force to have sex, 3) insisted on having sex, and 4) used threats to have sex.
**Nybergh study:** **(VAWI)**	My partner: 1) demanded to have sex with me even though I did not want to, 2) forced me to have sex against my will by using his/her physical strength, and 3) forced me to perform sexual acts that I experienced as degrading and/or humiliating.

Note: Our translation of items in NCK and extended NTU. To fit the table, some questions have been shortened. Original items and exact wordings can be found in each original study. In NorAQ and NCK, a question concerning who the perpetrator was is asked after the questions about each form of violent behavior.

**Table 6:** Methodology used in different prevalence studies of violence in Sweden. All are random population samples.

	NorAQ (new sample)	NCK study	Extended NTU	Lövestad study	Nybergh study
**Sample and response rate**	Women n = 749 (38%)	Women n = 5,681 (57%)	n = 12,671 (64.2%)	Women n = 282 (56.5%)	Women n = 624 (62.0%)
	Men n = 754 (35%)	Men n = 4,654 (47%)		Men n = 217 (43.5%)	Men n = 458 (45.5%)
Number included in analysis*	Women n = 749	Women n = 5,681	12,534	Women n = 251	Women n = 573
	Men n = 754	Men n = 4,654		Men n = 173	Men n = 399
**Age**	25–85	18–74	16–79	18–65	18–65
**Data collected**	2012	2012	2013	2009	2009
**Context of survey**	Focus violence. Not perpetrator-specific. Also includes questions about respondents’ health, stress and sense of coherence	Focus on violence. Not perpetrator-specific. Questions about health are included, and register data concerning health is also collected	Last part of a crime survey. Questions are framed as covering conflicts within an intimate partner relationship.	Focus on intimate partner violence (victimization, and perpetration).	Focus on intimate partner violence (victimization, and perpetration).
**Data collection method**	Web survey and postal questionnaire	Web survey and postal questionnaire	Telephone interview and Postal questionnaire	Postal questionnaire	Postal questionnaire
**Number of items**	Emotional: 3	Emotional: 4	Emotional:4	Emotional:5	Emotional: 4
	Physical: 2	Physical:5	Physical:2	Physical:12	Physical: 6
	Sexual: 4	Sexual:4	Sexual:2	Sexual:4	Sexual:3
**Instrument**	NorAQ. Validated in male and female sample using interviews as gold standard.	Own instrument, peer reviewed and tested in a pilot survey. Interviews with respondents to test the format.	Own instrument, peer-reviewed. Interviews with respondents to test the format.	CTS2, for sexual and physical violence and CBS, the Controlling Behavioral Scale.	VAWI. Psychometric properties tested among men and women in Sweden.

* Respondents were excluded due to not answering items concerning violence and/or if they had ever been in a relationship.

**Table 7:** Estimated prevalence rate (%) of different kinds of intimate partner violence in five different Swedish surveys.

	Women					Men				
	NorAQ	NCK	NTU	Lövestad	Nybergh	NorAQ	NCK	NTU	Lövestad	Nybergh
	New					New				
**Any violence**	16	N/A	26	N/A	N/A	5	N/A	17	N/A	N/A
**Emotional**	11	20	24	41	37	4	8	15	37	31
**Sexual**	5	7	N/A	N/A	N/A	0.3	1	N/A	N/A	N/A
**Physical**	10	14	N/A	N/A	N/A	1	5	N/A	N/A	N/A
**Sexual and/or physical**	12	N/A	15	27	24	2	N/A	8	21	14

Notes: N/A = Not Applicable, data were not presented in the respective studies.

Both Lövestad and Nybergh present data concerning past-year exposure and exposure earlier in life (before the past year). Lifetime prevalence rates have therefore been calculated from numbers given in Table 5 in Lövestad [28] and Tables 3 and 4 in Nybergh [27].

Prevalence rates in the NCK study only include violence after the age of 18 years.

## Discussion

The only evidence of nonresponse bias found was for differences between the sample and the background population concerning the sociodemographic characteristics. However, the magnitude of that effect is bleak in comparison with the differences found between the studies, e.g., prevalence of emotional violence for women: 11–41%, men: 4–37% and prevalence or sexual and/or physical violence for women: 12–27%, men: 2–21%. We found some potential explanations for the discrepancies in the prevalence rates between the studies, but unknown factors remain.

### Aim one: Nonresponse bias

The reported prevalence rate when using NorAQ was essentially replicated between the two samples. This was so despite the different response rates and time that had lapsed between the data collections. There was a higher prevalence of mild sexual violence in the new female sample. Likewise, in the last few years, the prevalence of sexual violence has increased in the annual Swedish crime survey, but not in the crime statistics or in the healthcare reports. It has been hypothesized that this may be due, in part, to the increased societal attention concerning sexual violence, making more victims prone to define their experiences as violence [35].

Young, unmarried, and foreign-born respondents were under represented in both the male and female new samples. These characteristics were associated with reporting violence; hence, it indicates an underestimation of the prevalence. However, the oldest men and women were also under represented but less likely to report all forms of victimization, indicating an overestimation of the prevalence.

We found different prevalence rates among those answering by paper and web, but the difference did not remain significant when controlling for age and sex. Hence, different modes of collecting data attracted different respondents but did not influence the result itself. Similarly, previous research found that using telephone, computer-based or paper-pencil format did not affect the prevalence rates of violence at significant levels [36, 37]. Wave analysis revealed differences in the background characteristics between the early and the late respondents but no difference concerning the reported prevalence rate. This is also consistent with previous research [3].

Violence can be considered a sensitive topic and hence, victims might be expected to be reluctant to participate [2, 22]. We found sensitivity of the subject to be an uncommon reason given for non-participation (2.7%). Among women, we found a tendency for victims to be more willing than non-victims to participate in future studies. Victims reporting none or moderate malaise when answering our survey were more willing to participate in a future questionnaire study, but victims reporting intense malaise were less inclined. This could indicate that victims, in general, are more motivated to participate, but that the most severely victimized are hesitant to participate. However, this aspect of the analysis approximates the nonresponse bias based on responders; hence, it may be misleading. It does concur with the hypothesis that victims subjected to the most severe forms of violence are less likely to respond to surveys; however, the group is rather small and hence does not affect the overall prevalence rates, to a great extent [9, 14, 38]. One previous study found that women who were physically abused by a male partner were more likely to participate in a survey than those who had not been abused. However, among severely victimized women, those living with their partner were less inclined to respond than those not cohabiting [39]. Instead, major reasons provided for non-participation in this study were survey overload and a general tiredness of surveys. This is consistent with previous findings [2].

### Aim two: Prevalence of intimate partner violence

The prevalence for intimate partner violence found in this study was markedly lower than in the other Swedish studies. Some of the discrepancies are easily understood while others are not.

There are considerable differences in the conceptualizations of emotional violence in the different instruments used (Table 5). In NorAQ, it is a prerequisite for emotional violence that it has been going on “systematically and for a long period of time.” The word ‘systematically’ is included in some of the questions in the NCK and the extended NTU but not at all in the Lövestad or Nybergh studies. “For a long time” is included only in the NorAQ. Hence, it is not surprising that the NorAQ produced the lowest estimate of emotional violence (women 11%, men 4%) followed by the NCK (women 20%, men 8%), and then the extended NTU (women 24%, men 15%), while considerably higher prevalence rates are reported both the Nybergh (women 37%, men 31%) and the Lövestad (women 41% men 37%) studies. In the Lövestad study, the highest prevalence rates given are for the items entitled: “wanted to know where I went and who I spoke to when not together” (women 28.3%, men 26.0%) and “felt suspicious and jealous of me” (women 27.1% men 27.2%) [28]. Even in a healthy relationship, it is not unreasonable to experience such feelings occasionally. Hence, for some respondents, a positive answer may not be indicative of controlling behaviors within a violent relationship. Analogously, some respondents include behaviors that do not occur in an aggressive context when reporting physical violence [10, 25].

Considering sexual and physical violence, the Lövestad and Nybergh studies again present considerably higher prevalence rates than the other studies, especially compared to the NorAQ study. One obvious difference between the studies is that we used an older population (age 25–85) compared to the Nybergh (age 18–65) and Lövestad (age 18–65) studies. Our own data (Table 3) as well as previous studies have found that younger people report higher prevalence rates. Also, mild forms of physical violence were included in all studies except the NorAQ; hence, the prevalence rate was expected to be lower in this study. However, in the old data collection (including the question about mild physical violence and using the age range 18–65), the prevalence of intimate partner physical violence was 13% for women and 3% for men, compared to 10% for women and 1% for men in the new data collection. Hence, neither the age range nor the mild physical item seem to be responsible for the much lower prevalence reported using the NorAQ, compared to the other studies. The reason the question about mild violence was omitted in the new data collection was that in the validation process, a considerable proportion of respondents subjected to mild physical violence did not relate to these experiences as abusive. This relates to findings concerning false positive, a possibility rarely considered in research on violence. It has been indicated that some respondents include entirely playful episodes such as pillow fights when responding to CTS2 (used in the Lövestad study), and potentially also being pushed or shoved in rush hour traffic [11]. Efforts to exclude violence occurring in a joking context in a survey involving youths had a significant impact on the prevalence rates [25]. What is considered a false positive depends on how violence is conceptualized, but a cognitive inconsistency can be found here. Violence occurring in a joking context is not part of what is generally considered intimate partner violence.

Differences concerning prevalence rates for physical violence may, in part, be explained by the number and specificity of items used. CTS2, used in the Lövestad study, includes the most items concerning physical violence: 12 compared to VAWI used by Nybergh (6 items), NCK (5 items), NorAQ (2 items), and the extended NTU (2 items). The items measuring behaviors that are not specified at all in the NorAQ (e.g., twisted arm or pulled hair, slammed against a wall, burned or scalded) did not yield very high prevalence rates in the Lövestad study [28]. Hence, the difference may not be attributed to the specific behaviors listed, but rather to the construction of questions. When several behaviors are included in each question as is done in the NorAQ, it might have affected the respondents’ understanding of the question. The same kind of questions are used in the NTU and to some extent also in the NCK, which produces a higher prevalence than the NorAQ, but lower than the Nybergh and Lövestad studies (Tables 5 and 7).

NorAQ has previously been compared to VAWI by Nybergh and colleagues [40, 41]. The questions in the NorAQ were then re-written to be partner-specific. Consistent with this study, the prevalence of emotional violence was found to be considerably lower when using the NorAQ compared to VAWI. However, differences in the prevalence rates concerning physical and sexual violence were not statistically significant. In this comparison, we found lifetime sexual and/or physical violence to be reported seven times more often by men and two times more often by women in VAWI compared to the NorAQ. One explanation for why the differences in Nybergh's studies were smaller can be that partner-specific wording was used in both instruments. Previously, the standard version of the CTS2, using partner specific language (e.g., my partner slapped me), was tested together with a revised version of the same instrument, using non-partner specific questions (e.g., has anyone slapped you followed by a question about who the perpetrator was). Gender symmetry, or more accurately higher prevalence rates for intimate partner violence among men than women, was found when using partner specific language (men 40%, women 33%). The non-partner specific version however produced the opposite result, gender asymmetry, with more women than men reporting intimate partner violence (men 24%, women 39%) [10]. The author hypothesized this to depend on the severity of the violence. Because men experience more severe forms of violence outside intimate relationships than women, it is possible that violence within intimate partnerships do not stay salient to them when prompted to think about other forms of violence as well. Women sustain more severe forms of violence within intimate partnership; hence, it will remain prominent to them also when asked about other forms of violence. None of the studies presented here found gender symmetry, but the differences in prevalence between the sexes are larger in the NorAQ and NCK than in Nybergh, Lövestad and extended NTU. This is in accordance with findings that using non-partner specific language produces gender asymmetry [10]. Though not presented here, both Nybergh and Lövestad found gender symmetry for past-year exposure to intimate partner violence, but not for exposure earlier in life [27, 28]. In their respective publications, they have similar reasoning for this as described concerning non-perpetrator specific language, i.e., because men are subjected to less severe violence in intimate relationships, it may not stay salient to them later in life.

The discrepancies in the prevalence rates are larger for men and greatest for emotional violence. The conceptualization of intimate partner violence stems largely from female victims, and violence has been found to be a gendered experience [42, 43]. The larger discrepancies concerning prevalence rates for men may indicate that the instruments are less precise when investigating male than female victimization and that there is a need to better conceptualize what intimate partner violence against men is.

How the survey is introduced may affect the results. In NorAQ, the study was framed as being about violence and ill-health, while the Nybergh study introduced the questions as concerning health, conflicts, and relationships. The extended NTU is part of a crime survey. However, the influence of introducing questions by saying they concern crime, health, or family has been reported to be modest [9].

In the NCK study, violence occurring before and after 18 years are presented separately. In our comparison (Table 7), only violence after 18 years is included. This could partly explain the lower prevalence found compared to Nybergh and Lövestad; however, NorAQ presents lifetime prevalence and reports even lower rates.

Another difference is the mode of collecting data. NorAQ, Lövestad, and Nybergh all used paper format, while NCK used the web and a paper format. NTU used telephone interviews and paper. However, in the analyses of nonresponse bias in this study, as in previous research, mode of inquiry did not affect the prevalence rates at significant levels [36, 37]. Extended NTU was the only survey which offers participants the opportunity to answer in languages beside Swedish which could potentially influence the prevalence rates, but the extended NTU reports prevalence in the mid-range so the effect does not seem to be considerable.

## Conclusions

The effect of nonresponse bias in our results was marginal compared to the discrepancies in the prevalence rates between the studies. We found some factors that partially explain the large differences. Most obvious were the diverging conceptualization of emotional violence and the effect of using partner specific or non-partner specific language. Because there is no universal definition of violence, it is reasonable to find discrepancies between the studies. However, it is not acceptable that so little is known about what produces these differences. In this study, we were not able to quantify the importance of different survey related factors. Some experimental research has been conducted to investigate the effect of different methodological approaches [1, 9, 10], but this study should be considered as a call for more empirical research concerning methods to investigate violence. Both quantitative and qualitative studies are needed. Scientific and technological innovations on measuring violence is essential for the reliability of future research in the field, especially when evaluating interventional studies.
